# The Impact of the Transition into First Employment on Smoking Behavior Among Young Workers in China

**DOI:** 10.3390/ijerph23040494

**Published:** 2026-04-14

**Authors:** Lingyun Meng, Yuxiao Hu, Jinqing Tao, Rong Zheng

**Affiliations:** 1 School of International Trade and Economics, University of International Business and Economics, Beijing 100029, China; 202200152004@uibe.edu.cn (L.M.); 202400130013@uibe.edu.cn (Y.H.); 202300130003@uibe.edu.cn (J.T.); 2 WHO Collaborating Center on Health Tax and Fiscal Policy, Beijing 100029, China

**Keywords:** young workers, smoking behavior, employment transition, China

## Abstract

**Highlights:**

**Public health relevance—How does this work relate to a public health issue?**

- Smoking remains a major public health burden in China, yet the smoking behavior of young workers during transition into first employment has received limited attention.
- First entry into the workforce exposes young individuals to new occupational stress and social norms that may promote increased smoking consumption.

**Public health significance—Why is this work of significance to public health?**

- Using nationally representative panel data and a staggered difference-in-differences design, this study shows that the transition into first employment is associated with increased smoking intensity among Chinese workers aged 16 to 35.
- The increase is associated with reduced life satisfaction and greater social engagement, while the transition into first employment is linked to lower smoking levels among older adults, indicating early career stages as a critical intervention window.

**Public health implications—What are the key implications or messages for practitioners, policy makers and/or researchers in public health?**

- Workplace tobacco control policies should pay particular attention to young workers entering their first job, including stronger enforcement of smoke-free regulations.
- Interventions such as smoking prevention programs integrated into workplace orientation or pre-employment health education may help reduce smoking escalation before habits become entrenched.

**Abstract:**

Existing research highlights the importance of young people in smoking prevention efforts, yet the smoking behavior of young workers remains underexplored. This study aims to examine whether the transition into first employment influences cigarette smoking among young Chinese workers and to explore the underlying mechanisms. Using data from the China Family Panel Studies (CFPS) and a difference-in-differences approach, we find that the transition into first employment significantly increases smoking intensity. Further analysis shows that this transition is associated with reduced life satisfaction, reflecting exposure to occupational stress such as high workload and time pressure, for which smoking may serve as a coping strategy. In addition, the transition into first employment is associated with increased drinking frequency, indicating greater social engagement in workplace settings where smoking and drinking are often embedded in social interactions. These findings suggest that tobacco control policies should target the first employment transition period by enforcing smoke-free regulations in workplaces and by integrating smoking prevention into pre-employment health education. Focusing on young workers during their first entry into the labor market offers a promising strategy to reduce future smoking prevalence in China.

## 1. Introduction

As the leading producer and consumer of cigarettes globally, China confronts a significant public health challenge. Smoking-related deaths increased by 57.9% from 1.5 million in 1990 to 2.4 million in 2019, representing the most substantial absolute rise globally [1]. Beyond its detrimental effects on individual health, smoking exerts a substantial economic strain on society, manifesting in escalated healthcare expenses and diminished workforce productivity. The World Health Organization estimated direct costs of tobacco-related diseases in China reached ¥53 billion (US $9 billion) in 2014, accounting for 1.5% of total healthcare expenditure [2]. Modelling by Yang Lian and her colleagues indicated smoking caused 7.67 million person-years of potential life lost (YPLLs) in 2014, averaging 15 years per smoking-related death. Consequently, the comprehension and mitigation of smoking habits have remained a paramount imperative within the realm of public health for an extended period.

Previous research has established that tobacco use initiation predominantly occurs among young individuals [3,4,5]. Behavioral and biological studies indicate that young people are particularly susceptible to addiction, with most adult smokers expressing regret about starting to smoke [6,7,8]. Research consistently shows that the majority of current smokers initiate tobacco use during adolescence or early adulthood, with studies estimating that 80% or more of smokers begin smoking between the ages of 14 and 25, and a significant proportion become regular smokers by age 15 [9,10,11]. However, studies also suggest that quitting smoking before the age of 35 can prevent many of its harmful consequences, with survival rates nearly matching those of individuals who never smoked [12,13]. Most young adult smokers begin as intermittent smokers but develop higher levels of addiction, eventually transitioning to regular smoking [14]. This explains why the tobacco industry not only targets young individuals to convert them into new smokers but also encourages intermittent smokers to become regular users [15]. This reality underscores the critical importance of focusing on young people in smoking prevention efforts. Without timely intervention, smoking behavior among young individuals can lead to long-term health consequences, thereby exacerbating the public health burden [16].

This study focuses on the smoking behavior of young workers during their transition into first employment for two primary reasons. First, the transition from campus to the workplace represents a critical period for the formation of smoking habits. During this phase, individuals move away from the supervision of schools and parents while facing occupational stress, defined here as the psychological strain resulting from high workload, time pressure, and limited autonomy, as well as societal expectations associated with environmental changes, leading them to seek relief through smoking [17,18,19]. Second, in the context of intense competition in the job market and the unique social connotations of smoking within Chinese cultural settings [20], young workers may feel compelled to smoke for social reasons. Existing research suggests that smoking employees under smoking managers may achieve higher positions [19].

Previous research has primarily focused on how unemployment increases smoking behavior [21] and the bidirectional relationship between employment and smoking. For instance, studies have shown that smokers are more likely to be absent from work, and take more sick leave annually [22] and that blue-collar jobs are associated with higher probabilities of obesity and smoking [23]. By focusing on young workers as a distinct population, this study investigates the impact of the transition into first employment on smoking behavior and explores several potential pathways that are indirectly associated with this transition, including occupational stress and social engagement. To isolate the influence of other factors on smoking initiation, this study also controls for individual characteristics such as income, thereby contributing to the existing body of literature.

According to the “Healthy China 2030” blueprint, China aims to reduce the smoking rate among individuals aged 15 and above to 20% by 2030. In recent years, China has implemented a series of measures to control smoking rates, but the task remains challenging. Data from the China Adult Tobacco Survey indicate that the smoking rate among individuals aged 15 and above was 28.1% in 2010 [24] and remained as high as 26.6% in 2018 [25]. While existing tobacco control measures have achieved some success, reducing the smoking rate by an additional 6 percentage points will require more effective policy interventions. Beyond tobacco taxation, it is crucial to focus on key populations and the critical stages during which smoking behavior is initiated.

In this study, we focus on workers aged 16–35, a cohort defined by China’s Labor Law, which prohibits the employment of individuals under 16, and by the common practice of many employers setting an upper age limit of 35 for recruitment. Our study aims to examine the impact of the transition into first employment on smoking behavior among young workers and to explore several potential pathways that are indirectly associated with this transition, including occupational stress and workplace social norms. The findings provide empirical evidence to inform the design of targeted early interventions and anti-smoking policies tailored to this population.

## 2. Literature Review

The multidimensional stress faced by young workers during career transitions is a significant factor contributing to smoking behavior. Studies have shown that high workload and low control in the workplace, such as repetitive tasks and a lack of decision-making authority, significantly increase the risk of smoking [26,27]. For instance, research on delivery riders in Guangzhou and Shenzhen, a representative emerging occupational group, reveals a nonlinear relationship between job-related uncertainty stress and smoking behavior. Moderate stress exhibits a protective effect, while extreme stress drastically increases the tendency to use smoking as a coping mechanism [28].

The symbolic meaning of tobacco, reinforced by social culture, becomes particularly pronounced in professional settings. In China, the prevalent phenomenon of “tobacco-based socialization” in the workplace has made smoking a crucial medium for young workers to integrate into groups and access resources. A survey conducted by the Shanghai Academy of Social Sciences found that 40% of adolescents initiated smoking due to peer pressure, such as being offered cigarettes by friends. Upon entering the workforce, this pattern evolves into a more complex dynamic involving power relations. Research indicates that smoking employees under smoking managers enjoy a 15% salary advantage compared to their non-smoking counterparts. This disparity is attributed to the “informal alliances” formed through shared smoking behaviors, which enhance managers’ performance evaluations of their subordinates [19].

## 3. Data and Methods

This study utilizes data from the 2010–2020 waves of the China Family Panel Studies (CFPS), a nationally representative survey conducted across 25 provinces, municipalities, and autonomous regions in China. Administered by the Institute of Social Science Survey at Peking University, the CFPS achieved a response rate of 84.14% and a cooperation rate of 87.14%. Using a multi-stage, probability-proportional-to-size sampling method, the CFPS covers approximately 95% of the Chinese population. The CFPS data are publicly available, anonymized, and were collected by Peking University with ethical approval from the university’s Institutional Review Board. As this study involves secondary analysis of de-identified data, additional ethical approval was not required.

We selected a sample of individuals aged 16–35 and processed the data as follows: (1) excluded observations with missing values for key variables. Missing values were handled by listwise deletion, as the proportion of missing observations was less than 3% for all included variables; (2) removed individuals who were continuously employed throughout the sample period to establish a clean control group, as our main analysis focuses on the transition into first employment. The treatment group consists of individuals who transition into first employment during the observation period, while the control group comprises individuals who remain non-employed throughout; and (3) applied 1% Winsorization at both ends to mitigate the impact of outliers. Winsorization at the 1st and 99th percentiles were applied to continuous variables (e.g., cigarettes, income) to reduce the influence of extreme values that could otherwise bias coefficient estimates. The final sample comprises 19,850 annual observations. Table 1 presents the summary statistics of the key variables for the final sample.

To empirically examine the impact of employment on smoking behavior among young workers, we employ a staggered difference-in-differences (DID) design estimated via a two-way fixed effects (TWFE) model. This approach compares the change in smoking behavior among the treatment group around their first employment event with the concurrent change among the control group, thereby netting out time trends and time-invariant differences between the two groups. The model is specified as follows:

(1) $${Cigarettes}_{it}=\alpha +{\beta Employ}_{it}+\gamma Controls+\mu_{i}+\lambda_{t}+\varepsilon_{it}$$

In the model, the subscript i represents the individual, and t represents the year. The dependent variable ${Cigarettes}_{it}$ indicates the daily number of cigarettes smoked by individual i in year t, based on the survey question “On average, how many cigarettes do you smoke per day?”. Non-smokers are coded as 0. The key explanatory variable ${Employ}_{it}$ represents the employment status of individual i in year t, taking a value of 1 if employed and 0 if unemployed, derived from the question “Did you have a job in the past 12 months?”. Controls represents a series of individual- and household-level control variables. The individual fixed effects, $\mu_{i}$, are included to control for time-invariant factors at the individual level. To account for the effects of macroeconomic cyclical fluctuations, year-fixed effects $\lambda_{t}$ are also controlled for. Additionally, $\varepsilon_{it}$ is the random error term, and robust standard errors clustered at the individual level are used.

## 4. Results

### 4.1. Baseline Regression

Table 2 reports the baseline results. Column (1) indicates a significant positive correlation between transition into first employment and smoking intensity among young workers. Columns (2) and (3) sequentially introduce individual-level and household-level control variables, and the previously observed results remain robust. The coefficient for smoking intensity in Column (3) suggests that, after entering first employment, young individuals increase their daily cigarette consumption by 0.44 cigarettes on average.

### 4.2. Parallel Trend Test

**Figure 1:**
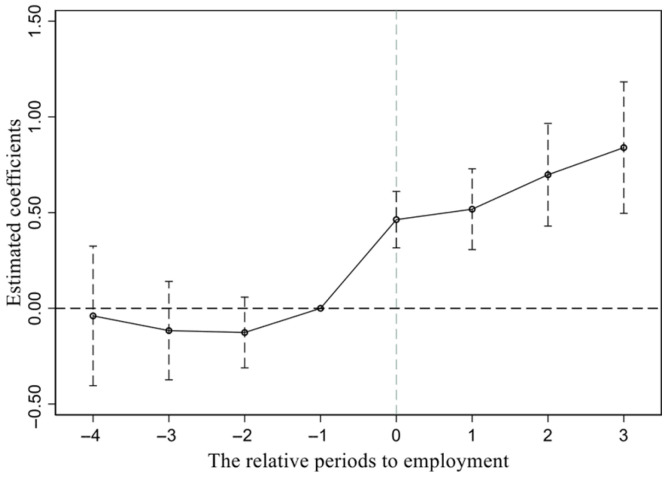
Parallel trend test.

A key prerequisite for applying the Difference-in-Differences (DID) model is the satisfaction of the parallel trend assumption, which requires that the smoking behaviors of the treatment and control groups follow a similar trend prior to employment. To test this assumption, an event study approach was employed. In Figure 1, the coefficients for pre1 to pre4 are not statistically significant, indicating no differences between the treatment and control groups before employment, consistent with the parallel trend assumption. Additionally, the positive and significant coefficients for smoking behavior after the transition into first employment further corroborate the baseline regression results.

### 4.3. Heterogeneous Treatment Effects

**Figure 2:**
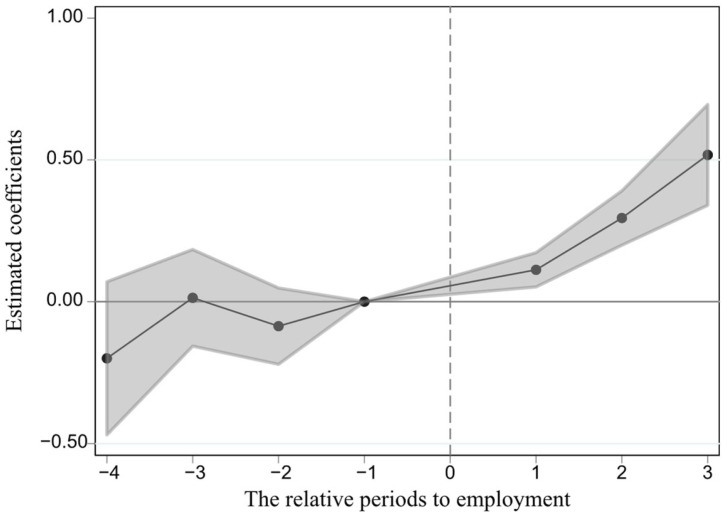
Heterogeneous treatment effects test.

Due to variations in employment timing among individuals in the treatment group, those who enter employment earlier may serve as the control group for those who are employed later, leading to estimation bias in the traditional Two-Way Fixed Effects (TWFE) model due to the presence of “bad controls” [29,30,31]. To address this issue, the study re-examines the parallel trends assumption using the improved method proposed by Sun and Abraham and presents the results in the form of an event study [29]. As shown in Figure 2, the coefficients for the increase in smoking behavior post first employment remain significantly positive, indicating that the “negative weights” associated with heterogeneous treatment effects in staggered DID do not undermine the robustness of the baseline regression results.

### 4.4. Robustness Tests

First, considering that the increased smoking intensity may be due to the income effect resulting from an increase in personal income levels after the transition into first employment, this study further includes the logarithm of individual income as a control variable to block the channel through which changes in individual income due to first employment may affect smoking intensity. The results in column (1) of Table 3 show that the baseline results remain robust after controlling for changes in individual income.

In addition, to reduce sample selection bias and ensure balance in characteristics between groups, the Propensity Score Matching Difference-in-Differences (PSM-DID) method was employed to re-estimate the Model (1). The results, presented in Column (2) of Table 3, are consistent with the baseline findings. Additionally, when the dependent variable was replaced with the smoking rate, as shown in Column (3) of Table 3, the results remained robust.

Furthermore, to explore whether the increased smoking rate and smoking intensity associated with the transition into first employment vary across different age groups, we further conducted regressions on the two dependent variables for individuals over 35 years old. The results, presented in Columns (4) and (5) of Table 3, show that the coefficients for first employment are significantly negative, indicating that while young individuals tend to increase smoking after their first employment, middle-aged and older individuals reduce their smoking behavior upon their first employment. This finding aligns with previous research suggesting that unemployment increases smoking behavior [21]. As a further robustness check, we re-estimated the model using the full sample that includes continuously employed individuals. The results, shown in Column (6) of Table 3, remain consistent with the baseline findings, confirming that our conclusions are not driven by sample selection.

### 4.5. Mechanism Analysis

During the transition from school to the workplace, young workers may experience occupational stress, difficulties in adapting to new roles, and changes in social interaction patterns, which may lead them to start smoking or increase their smoking intensity. In particular, for non-smokers, refusing cigarettes can cause discomfort or even embarrassment, as Chinese culture often views the repeated offering of cigarettes as a gesture of hospitality. Smoking, therefore, serves as a social lubricant, facilitating daily interactions with bosses, colleagues, and potential relationships [19,20].

We explore whether the transition into first employment is associated with occupational stress and social engagements among young workers. Due to data limitations, directly measuring social engagement is challenging. Although job satisfaction is available in the CFPS as a potential indicator of occupational stress, it is only measured for employed individuals and therefore cannot capture changes around the first employment transition within our fixed-effects framework. We therefore use available proxies: life satisfaction as an indirect indicator of occupational stress, and drinking frequency as a proxy for social engagement. Columns (1) and (2) of Table 4 show that this transition is associated with increased drinking frequency and reduced life satisfaction among young individuals. The results in Column (3) are not statistically significant, suggesting that the transition into first employment does not significantly affect their confidence in the future.

Additionally, Table A2 presents a heterogeneity analysis based on gender, educational attainment, and the proportion of smokers within the household. First, we find that the transition into first employment significantly increases smoking intensity among young men but has no significant effect among young women. Second, individuals with lower educational attainment exhibit a larger increase in smoking intensity after transitioning into first employment compared to those with lower educational levels. Finally, young workers from households with a higher proportion of smokers show a significant increase in smoking intensity after entering first employment.

## 5. Discussion

This study provides empirical evidence that the transition into first employment is associated with increased smoking intensity among young workers in China. Using nationally representative panel data and a TWFE model, we find that this transition is associated with an average increase of 0.44 cigarettes per day. This finding remains robust after a series of sensitivity checks.

Our findings align with prior research documenting that unemployment increases smoking behavior [21], but extend this literature by showing that the transition into first employment, rather than unemployment, represents a critical period for smoking escalation among young workers. This is consistent with developmental frameworks that highlight early career stages as periods of heightened vulnerability to health risk behaviors [18]. The observed increase in smoking intensity during the first employment transition suggests that workplace entry may serve as a risk environment where smoking habits are formed or intensified, a dimension that has received limited attention in previous studies. The practical significance of an increase of 0.44 cigarettes per day should be considered in the context of population health. While modest at the individual level, this increase, if sustained, could translate into substantial cumulative health burdens given the large number of young workers entering the labor market each year in China.

The effect of the first employment transition on smoking is not uniform across subgroups. The increase is concentrated among young men, a pattern that may reflect the greater social acceptance of smoking among men in Chinese culture [32]. In addition, young workers with lower educational attainment show a larger increase in smoking intensity after entering first employment, a finding consistent with Pan (2004) [20]. This may be explained by the fact that better-educated individuals are more frequently exposed to smoking prevention education and monitoring in both school and workplace settings. Furthermore, young workers from households with a higher proportion of smokers exhibit a stronger increase in smoking intensity, which may be attributed to their higher acceptance of smoking behavior, making them more likely to consume more cigarettes [33]. Collectively, these patterns suggest that the social environment, both at home and in the workplace, plays a role in shaping smoking behavior during the transition into first employment.

The exploratory mechanism analyses suggest that the transition into first employment is associated with occupational stress, as reflected in reduced life satisfaction, as well as with increased social engagement measured by drinking frequency. These patterns are consistent with the idea that both psychological strain and workplace social norms may contribute to smoking escalation. However, due to data limitations, these mechanisms remain suggestive rather than definitive. Direct measures of workplace stress, peer smoking behavior, and social norms are needed in future research to further clarify these pathways.

The findings highlight the importance of targeting the first employment transition period for tobacco control interventions. The observation that smoking intensity increases upon first entry into the labor market suggests that this period constitutes a critical window for prevention efforts. Interventions directed at this transition point may prove more effective than those implemented later, after smoking habits have become established.

## 6. Conclusions

This study examines the association between the transition into first employment and smoking behavior among young workers in China using data from the 2010 to 2020 waves of the CFPS. The results show that the transition into first employment is significantly associated with increased smoking intensity among individuals aged 16 to 35. This finding remains robust after a series of sensitivity checks. The study also explores potential mechanisms and finds that the transition into first employment is associated with reduced life satisfaction, and increased social engagement, patterns consistent with stress-based and social-based explanations.

From a policy perspective, and given the associational nature of our evidence, the following implications should be considered tentative and hypothesis-generating rather than conclusive. Nevertheless, they point to potential intervention opportunities that merit further investigation. Workplace smoke-free regulations could be considered for stricter enforcement, particularly in settings where young workers are first employed. In addition, interventions such as smoking prevention programs integrated into workplace orientation or pre-employment health education may be beneficial in reducing smoking escalation before habits become entrenched. As China pursues the Healthy China 2030 smoking reduction target, focusing on young workers during their first entry into the labor market represents a potentially promising strategy, though further research with stronger causal identification is needed to confirm these findings.

## 7. Limitations

Despite its contributions, this study has several limitations. First, due to data constraints, we are unable to explore the heterogeneity of smoking behavior across different occupations. The impact of the transition into first employment on smoking may vary significantly depending on the nature of the job, such as the level of occupational stress, workplace culture, and social dynamics specific to certain industries. Second, our mechanism analysis relies on indirect proxies rather than direct measures. We use drinking frequency as a proxy for social engagement and life satisfaction as a proxy for occupational stress. Direct measures of workplace social norms, peer smoking behavior, or validated stress scales are not available in the CFPS, limiting our ability to draw causal conclusions about mechanisms. Future research with richer occupational and psychosocial data is needed to further explore these pathways.

## Figures and Tables

**Table 1:** Summary statistics.

	Mean	SD	Min	Max
Cigarettes	1.883	5.011	0.000	20.000
Employ	0.452	0.498	0.000	1.000
Marry	0.558	0.497	0.000	1.000
Eduy	11.118	4.086	0.000	35.000
Health	2.436	1.013	1.000	5.000
Exercise	1.768	0.783	1.000	3.000
lnFamincnet	10.578	1.183	0.000	16.156
HHcursmoker	0.837	0.791	0.000	6.000
HHSpp	0.316	0.247	0.000	1.000
Familysize	4.548	1.948	1.000	17.000

The total number of observations is 19,850.

**Table 2:** Baseline results.

	Cigarettes		
	(1)	(2)	(3)
Employ	0.5902 ***	0.5989 ***	0.4411 ***
	(0.0764)	(0.0764)	(0.0730)
Individual FE	YES	YES	YES
Year FE	YES	YES	YES
Time-varying individual controls	NO	YES	YES
Time-varying household controls	NO	NO	YES
Observations	19,850	19,850	19,850
R^2^	0.7905	0.7909	0.8088

*** indicate significance at the 1% level.

**Table 3:** Robustness tests.

	(1)	(2)	(3)	(4)	(5)	(6)
	Smoking Intensity	PSM-DID	Smoking Rate	Individuals over 35 Years Old		Add Continuously Employed Individuals
		Smoking Intensity		Smoking Intensity	Smoking Rate	Smoking Intensity
Employ	0.3396 ***	0.6060 ***	0.0240 ***	−0.2412 **	−0.0123 ***	0.3273 ***
	(0.0775)	(0.1617)	(0.0047)	(0.1111)	(0.0046)	(0.0712)
Individual FE	YES	YES	YES	YES	YES	YES
Year FE	YES	YES	YES	YES	YES	YES
Observations	19,850	5484	19,161	26,942	26,942	37,644
R^2^	0.8091	0.8061	0.8893	0.8339	0.8867	0.8343

*** and ** indicate significance at the 1% and 5% levels, respectively.

**Table 4:** Mechanism analysis.

	(1)	(2)	(3)
	Drink	Life Satisfaction	Confidence in the Future
Employ	0.0152 ***	−0.1764 ***	0.0275
	(0.0055)	(0.0289)	(0.0259)
Individual FE	YES	YES	YES
Year FE	YES	YES	YES
Observations	19,850	13,556	13,555
R^2^	0.5292	0.5601	0.5650

*** indicate significance at the 1% level.

## Data Availability

The original data presented in the study are openly available in the China Family Panel Studies (CFPS). The data can be accessed by registering at https://cfpsdata.pku.edu.cn/. Restrictions apply to the redistribution of this data, as per the CFPS user agreement which prohibits users from depositing the data or any derived datasets on any third-party platform.

## Abbreviations

The following abbreviations are used in this manuscript:

DID	Difference-in-Differences
CFPS	China Family Panel Studies
PSM-DID	Propensity Score Matching Difference-in-Differences

## Appendix A

### Appendix A.1

Variables definitions are given in Appendix A Table A1.

**Table A1:** Variable definitions.

Variable	Definition
Cigarettes	Daily number of cigarettes smoked
Employ	Dummy variable: 1 if currently employed, 0 otherwise
Marry	Dummy variable: 1 if married, 0 otherwise
Eduy	Years of education completed by the respondent
Health	Self-rated health of the respondent on a scale from 1 to 5:1 for excellent, 5 for poor
Exercise	Dummy variable: 1 if exercises regularly, 0 otherwise
lnFamincnet	Logarithm of household net income
HHcursmoker	Number of individuals in the household who currently smoke
HHSpp	Proportion of individuals in the household who currently smoke
Familysize	Household size
Smoker	Dummy variable: 1 if smoker, 0 otherwise
Gender	Dummy variable: 1 if man, 0 otherwise
Alcohol	Dummy variable: 1 if drinks alcohol three or more times per week, 0 otherwise
Lifesatisfy	Satisfaction with life on a scale from 1 to 5:1 for very dissatisfied, 5 for very satisfied
Futurecon	Confidence in the future on a scale from 1 to 5:1 for very unconfident, 5 for very confident

### Appendix A.2

Table A2 presents a heterogeneity analysis based on gender, educational attainment, and the proportion of smokers within the household.

Regression analysis is conducted based on groups divided by the median of individual years of education and the proportion of smokers in the household.

**Table A2:** Heterogenous analyses.

	Gender		Educational Attainment		The Proportion of Smokers Within the Household	
	Woman	Man	Low	High	Low	High
Employ	0.0216	0.9484 ***	0.5875 ***	0.3161 ***	0.0739	0.7092 ***
	(0.0280)	(0.1706)	(0.1100)	(0.0920)	(0.0514)	(0.1338)
Individual FE	Yes	Yes	Yes	Yes	Yes	Yes
Year FE	Yes	Yes	Yes	Yes	Yes	Yes
Observations	11,115	8697	11,907	7943	9929	9921
R^2^	0.6357	0.7988	0.8241	0.7461	0.6972	0.8045

*** indicate significance at the 1% level.
